# Stress, migration, and allostatic load: a model based on Mexican migrants in Columbus, Ohio

**DOI:** 10.1186/s40101-018-0188-4

**Published:** 2018-12-13

**Authors:** Alexandra C. Tuggle, Jeffrey H. Cohen, Douglas E. Crews

**Affiliations:** 1 Department of Anthropology, Ohio State University, 4034 Smith Laboratory, 174 W. 18th Avenue, Columbus, OH 43210 USA; 2 College of Public Health, Ohio State University, 250 Cunz Hall, 1841 Neil Avenue, Columbus, OH 43210 USA

**Keywords:** Acculturation, Biomarkers, Health, Immigration, Stressors

## Abstract

**Background:**

Immigration is a disruptive event with multiple implications for health. Stressors, including family separation, acculturation, job insecurity, restricted mobility, sojourns, dangerous border crossings, stigmatization, and marginalization, shape immigrant health in ways we are only beginning to untangle. Around the world, there are over 200 million international migrants. In 2015, there were 43.2 million immigrants living in the US, 26.8% of whom were born in Mexico. Investigating how stress affects health among migrants facilitates better understanding of their experiences.

**Methods:**

Here, we review existing research on stress and how allostatic load varies among migrants with specific attention to Mexican migrants in the US. Next, we explore research incorporating biomarkers of allostasis and narratives of migration and settlement to examine disease risks of Mexican migrants residing in Columbus, Ohio. This mixed-methods approach allowed us to examine how social stressors may influence self-reports of health differentially from associations with assessed discrimination and physiological biomarkers of health.

**Results:**

These data sources are not significantly associated. Neither narratives nor self-reports of health provide significant proxies for participants’ physiological health.

**Conclusions:**

We propose, the pairing of objectively assessed health profiles with narratives of migration better illustrate risks migrants face, while allowing us to discern pathways through which future health challenges may arise. Immigration and acculturation to a new nation are biologically and culturally embedded processes, as are stress and allostatic responses. To understand how the former covary with the latter requires a mixed-methods bioethnographic approach. Differences across multiple social and physiological systems, affect individual health over time. We propose incorporating physiological biomarkers and allostatic load with migrants’ narratives of their migration to unravel complex relationships between acculturation and health.

## Background

The deterioration of immigrant health following migration is closely intertwined with stressors they experience before, during, and after their journeys. Transitioning into a new society and the social adjustment that follows produce unique stressors for migrants. For all species, their environment is a significant stressor, and any change in that environment may have a corresponding effect on health. As such, all organisms have evolved in some way to halt and delay stressors long enough to reproduce [1]. Allostasis theory was developed to help explain this general mammalian life history trait, allocating energy for physiological stress responses to limit somatic wear and increase an organism’s survival and reproduction [2]. In their original conception, Sterling and Eyer [2] defined allostasis as “stability through change,” highlighting the dynamic character of internal mammalian physiological systems evolved to react to their continuously fluctuating activity levels and environmental conditions (p. 636). Although dynamic short-term physiological adaptability to stressors is beneficial to organisms during everyday life, such responsiveness and flexibility come at a cost. Over time, activation of allostatic mechanisms in response to stressors results in cumulative wear and tear. This long-term accumulation of somatic damage is an allostatic load. As allostatic load increases, it may manifest as chronic disease following deterioration of internal regulatory systems, cognitive function, and physical performance [3–5].

Stressors abound in everyday life, and some are more likely to instigate chronic allostatic responses than others. For example, migration is a significantly disruptive life event that impacts multiple dimensions of health. Chronic exposures to stressors associated with migration may take their toll on migrant health and lead to multisystem physiological dysregulation, or allostatic load. Hardships before and during migration, combined with trials while acculturating to a new society, may substantially disrupt an individual’s life manifesting as higher allostatic load. Estimates of allostatic load are associated significantly with morbidity, mortality, and declines in physical and cognitive function among samples of older adults across populations [6–9]. Because allostatic load is cumulative over time, negative health outcomes resulting from chronic exposures to stressors may be disproportionately expressed by elders.

We begin with a review of existing research on stress responses and the effects of stressors on the health of international migrants. While extensive research has examined relationships of stressors and stress to the migrant experience, only recently have migration researchers utilized biomarker assays of physiological function to assess stress-related physiology and allostatic load. A majority of early research approached migration-related stress by focusing on proposed measures of acculturation and the “healthy immigrant effect.” The healthy immigrant effect describes the phenomenon wherein immigrants experience a health advantage compared to native-born individuals that declines with time spent in the destination country [10]. More recent research on migrant health has incorporated assessments of allostatic load into research design [11–16]. Next, we report a specific example wherein biomarkers of allostasis, self-reports of health, and narratives of migration and settlement were examined in a sample of Mexican migrants residing in Columbus, Ohio. We use these data and analyses to propose a model for the multiple pathways we observe linking migration, perceptions of health, and stressors. Following this model may help us, and others, continue testing the complex and competing social, ideational, and physiological stressors, and possible outcomes migrants experience during their settlement.

Latinos are the second fastest growing minority group in the US. In 2015, there were 43.2 million immigrants living in the US, 26.8% of whom were born in Mexico [17]. Research documenting how migrants adapt to stressors, subsequent internal stress, and related physiological dysregulation leads to deteriorating health of migrants is timely as around 244 million people worldwide currently are migrants [18]. This research also supports achieving goals of Healthy People 2020 [19] and reducing racial and ethnic health disparities within the US. During the current period of heightened anti-immigrant sentiment worldwide, it is more pertinent than ever to acknowledge hardships experienced by a large and growing proportion of many populations (e.g., economic, climate, and refugee migrants).

The decision to migrate in the first place is often driven by insecurity in the home country. This complex decision may be influenced by stressors such as conflict, economic hardships, political instability, and trauma at the point of origin. It may then be followed by stressors stemming from unanticipated problems at the point of destination [20, 21]. For those who arrive on foot, the journey across the US southern border is physically and mentally demanding [22]. However, physical stressors are but one of many. Exposures to poor living conditions in their home country prior to deciding to migrate, along with separation from their family and local support systems play an important role in migrants’ response to stressors during their sojourns. After arriving at their point of destination, migrants face additional stressors that may include entry/passport control, documentation status, finding a stable local setting, job insecurity with often low-paying, physically demanding work, restricted mobility due to fear of deportation or inability, stigmatization, and discrimination by the local population, and marginalization, as well as long-term acculturative stressors [23, 24]. Additional stressors of acculturation include language difficulties, age at arrival, and adoption of unhealthy lifestyles [11, 25, 26]. All may heighten chronic stress and thereby shape immigrant health in complex ways continuing research is only beginning to untangle.

We propose that by pairing objective health profiles with narratives of migration and self-reports of health, we will better capture risks faced by migrants and discern interconnecting pathways that result in their greater health challenges. Because immigration, acculturation, and health are biologically and culturally embedded, they must interactively affect health. Examining one without assessing the other provides a one-sided and likely inaccurate picture of current and future health risks.

### Acculturation, health, and the “Healthy Immigrant Effect”

Immigrant populations are naturally diverse. Therefore, researchers have studied immigrants from multiple perspectives [27]. Acculturation models are used frequently in other minority health research [26], but are the most common framework in migrant-specific health research. Acculturation, or processes of cultural adjustment and acclimatization by migrant populations to their destination country, are important influences on health and health disparities [28–32]. However, the reported ways acculturative factors affect immigrant health are not consistent throughout the literature [32–36] and appear to differ across migrant populations examined, as the reality of migration is different for everyone. Stress results from an individual’s perception and appraisal of their current life circumstances. For example, acculturative stressors may reflect migrants’ separation from their family and friends, need to learn a new language, adapt to new and different cultural behaviors, and enter a new workforce, and sometimes problems with legal documentation. Since physiological responses to stressors are so closely associated with individual and cultural perceptions, it might be expected that acculturative stress research would yield inconsistent conclusions as to influences of these factors on health across study populations.

One theme widely recognized across this research is the healthy immigrant effect. The healthy immigrant effect was based on findings that immigrants to the US experienced a health advantage compared to US-born individuals [10], an advantage that declined with time spent in the US. This commonly is hypothesized to represent positive selection for pre-migration health—only the healthiest undertake the journey that marks successful immigration [25, 32, 37]. The associated health decline most often is attributed to assimilation of unhealthy behaviors associated with acculturative stressors, usually smoking or drinking [38, 39], worsening diet, and decreased physical activity [25]. Additional stressors influencing health disparities among migrants are discrimination and legal concerns [23, 24, 40].

In a 2009 paper, Finch and colleagues [10] tested a new model of immigrant health called the “Oliver Twist theory,” a variation of the healthy immigrant effect. This theory posits immigrants moving from less-developed countries to more socioeconomically advantaged ones initially will show health advantages due to a lower infectious disease load and lower infant mortality at their destination. However, their advantage declines as they adapt to or adopt local cultural patterns and practices that may promote chronic disease. Using NHANES data, Finch et al. [10] examined four groups: non-Hispanic white Americans, US-born Mexican Americans, recent Mexican immigrants, and long-term Mexican immigrants using three different outcomes: self-rated health, physician-reported health, and allostatic load (based on Seeman et al.’s [5] 10-biomarker index, but using clinically defined high-risk cut points rather than quartiles). As a general pattern they observed, recent immigrants exhibited the best health, followed by long-term immigrants, non-Hispanic whites, and US-born Mexican Americans, who exhibited the poorest overall health. Recent immigrants were healthier across all three health assessments and for all biomarkers of allostatic load, excepting inflammatory biomarkers. Smoking was a significant influence on health, while the estimated proportion of smokers was lowest in more recent immigrants.

Riosmena et al. [34] investigated the relationship between time spent in the US and poorer health outcomes as a problem with negative acculturation, suggesting most measures of acculturation used to explain health disparities between migrants and natives neglect important structural aspects of the process. First and foremost, acculturation is a process, not an event. Migrants are continually acculturating, and different types of assessments may reflect different patterns and pacing thereof. Riosmena et al. [34] suggest cross-sectional sampling common to migrant health research mask experiences of poverty and hardship prior to migration. When assessing negative acculturation as a determinant of negative associations between mortality and length of residence in the US, Riosmena et al. [34] estimated acculturation using duration of residence in the US, English language preference, legal status, and sex differences, along with additional factors.

In this sample of 80,472 Latinos residing in the US from the National Health Interview Survey (NHIS) from 1998 to 2006, among both sexes, English language proficiency and, for women, acquired citizenship are associated with better health [34]. Their results suggest acculturation is neither the sole nor even the main explanation for negative association between duration of stay in the US, greater chronic disease, and poorer survival. Although accounting for behavioral differences, body mass index (BMI), and socioeconomic status (SES), their use of mortality data may influence conclusions as there are constraints on mortality reports. Death is a hard endpoint, but potential for bias remains. For example, return migrants may be the least healthy of those who arrived, possibly biasing mortality estimates. Similarly, less acculturated migrants likely are more likely to return home when ill or they have failed to succeed.

Arbona et al. [23] explored unique stressors disparately affecting documented and undocumented Latino immigrants to the US. Undocumented migrants experience more immigration-related stressors, for example, separation from their family, traditionality, language difficulties, and legal issues. However, both groups experienced similar levels of deportation fear. Arbona et al. [23] also investigated differences in effects of extrafamilial (e.g., occupational, immigration, legal) and intrafamilial (e.g., marital, parental, cultural, family) stressors. Both immigration stressors and legal status were uniquely associated with extrafamilial factors. Fear of deportation commonly predicted both extrafamilial and intrafamilial stressors across all groups regardless of documentation, making it a poor proxy for legal status. Additionally, a sex difference was revealed in experienced stressors. Women were more likely to be documented. Thus, men, being more often undocumented, were more susceptible to extrafamilial stressors such as separation from family and occupational stressors, suggesting their possible greater stress and physiological dysregulation.

To investigate the connection between self-rated health, acculturation, and health inequalities, Todorova et al. [36] sampled 1357 Puerto Rican immigrants to Boston, mean age 57.2. More successfully acculturated respondents rated their health more positively. Self-reports of better health also correlated with better indicators of assessed psychological and physical health. Low self-reported health was associated with being female, low emotional support, engaging in risky behaviors, and poverty. Self-reported health differed by level of acculturation, and the less acculturated tended to minimize their perceived health problems. Todorova et al. [36] concluded self-reports of health may be inaccurate and even mask health disparities and related contributing factors, an important consideration in our proposed model.

Another approach to migrant health research uses age at migration as a factor in the relationships between migration stressors and health outcomes. In a sample of 2848 Mexican Americans (immigrants and US-born) in the US, Angel et al. [41] used proportional hazard models to assess differential mortality risk correlated with age at migration. They found that “immigrant” does not constitute a collective risk category. Those who migrated at younger or middle ages experienced similar mortality as did US-born Mexican Americans. Conversely, those who migrated during late life experienced a considerable mortality advantage. This may be due to different qualities of migrants in each age group. The youngest migrants generally are selected based on their parents’ characteristics. Parents or middle-aged adults who migrate to the US are generally able-bodied, healthy individuals who find work in service sector jobs that are physically demanding and often lack health benefits. Earlier age at migration also results in longer exposure to negative acculturative processes. Older migrants come to the US for different reasons and thus represent a unique risk group. Migrants over age 50 rarely come seeking work opportunities and generally come to reunite with family. Although they are significantly disadvantaged in education, a familial source of social support and the possibility of living in a healthier environment apparently lead to significant mortality advantages among older migrants.

### Incorporating biomarkers of allostatic load

More recently, migrant researchers have incorporated measures of allostatic load into their research designs. Indicators of acculturation are correlated with stress, but are subjective and vary widely based on subgroup characteristics. Allostatic load indices have the distinct advantage of being relatively unbiased in revealing the physiological dysregulation that is associated with chronic stressors. In a landmark study, Kaestner et al. [14] tested the “unhealthy assimilation” hypothesis to explain worsening health of Mexican immigrants to the US using allostatic load. Their results agreed with the healthy immigrant effect. Mexican immigrants often are healthier upon arrival than US-born Mexican Americans. However, they also experience health declines in a direct association with time they spend in the US. While stress is cited as the cause of such health disparities, Kaestner et al. [14] measured allostatic load, a health outcome believed to be directly associated to chronic stress, rather than relying on participants’ subjective self-assessments. Allostatic load increased significantly with time living in the US among 2459 Mexican Americans aged 45–60 years, supporting the healthy immigrant effect.

In a 2010 paper, Peek and colleagues [16] investigated allostatic load among white Americans, African Americans, and people of Mexican origin (immigrants and US born) to examine the influence of ethnicity and acculturation on health disparities. Using a 12-biomarker allostatic load index and a verbal interview to measure acculturation, they observed a pattern similar to that hypothesized by the healthy immigrant effect. Interestingly, Peek et al. [16] also observed Mexican immigrants had lower allostatic load than “white” Americans, but that there was no association of acculturation with allostatic load. This is surprising as previous research suggested migrants living in the US for 10+ years exhibited poorer health than observed among more recent immigrants. They did not however explore age and sex differences in their analysis. The “healthy migrant effect” implies more acculturation is damaging to immigrants’ health. However, Peek et al.’s [16] analyses do not support such an association.

While most of the previously detailed research relies on nationally representative datasets (e.g., NHANES), smaller regional samples are effective at detecting small-scale local patterns in migrant health that may be masked by larger samples. In a study of 238 African immigrants to the US, Bingham et al. [12] utilized a 10-biomarker index to examine associations between allostatic load, age of immigration, reason for immigration, and unhealthy assimilation behavior. They found that overall, African immigrants are not likely to develop unhealthy assimilation behaviors such as alcoholism, smoking, and decreases in their physical activity. They further reported, although older age of immigration and a longer stay in the US were associated with higher allostatic load, there was no association with region of origin, education, gender, nor income. They also reported that among all possible migration reasons, family reunification was associated with the lowest allostatic load scores. Bingham et al. [12] also posited that a large proportion of their sample self-identified strongly as African; therefore, they adhered largely to their birth country traditions and were less likely to acquire unhealthy behaviors as a result of acculturation.

McClure et al. [15] investigated associations between allostatic load, perceived stress, and settlement community context among Mexican immigrant farmworkers in a small Oregon community (*N* = 126). Their sample included two different social settings: one, a majority Mexican immigrant enclave and, two, “White” English-speaking residential areas. While migrant groups in both settings exhibited higher allostatic load, social stressors and buffers differed by community. Low family support, a stressor generally thought to be associated with high allostatic load among Mexican immigrants, was associated significantly with allostatic load only among Mexican women residing in the White-majority communities. Suggesting, women in Mexican-majority communities may experience greater social buffering from stressors associated with residence in the US. Additionally, women residing in the Mexican enclave reported lower discrimination-based stressors than their counterparts in predominantly white communities. However, women’s allostatic load scores did not differ significantly between sites. Such results support a model that stressors perceived and narratives of informants are not necessarily reflective of their physiological responses or their measurable biomarkers in a linear fashion, nor do they parallel each other. Although theirs was a small sample (*N* = 126) compared to research utilizing large, nationally representative databases like NHANES, these analyses provide insights into the complex interactions between biology, culture, psychology, and stressors. Regional research, such as that of McClure et al. [15], Bingham et al. [12], and Peek et al. [16], provide a basis for suggesting research utilizing large, nationally representative samples may mask local patterns and variable responses to stressors.

### A model based on Mexican immigrants to Columbus, Ohio

Columbus, Ohio, is not a traditional destination for Mexican immigrants to the US; however, the city’s Mexican community has grown rapidly over the past 20 years with some long-term residents having arrived over 40 years ago [42]. This long-term occupation and relatively large population (*N* = 67,000) [43] makes Columbus an ideal setting to explore models linking migration and health with allostatic load. Here, we develop a model incorporating assessments of allostatic load with cultural narratives of migration and self-reports of health, based on interviews with Mexican migrants in Columbus, Ohio. Data were obtained across multiple domains with mixed-methods including interviews, narratives of migration, self-reports of health, self-reported discrimination, and assessments of several biomarkers often used to estimate allostatic load. Narrative and interview data were obtained from 34 individuals, of whom 28 participated in biometric measurements. Our original hypothesis was that measures of allostatic load would be positively associated with self-reports of health and discrimination, as well as reflective of participants’ narratives of migration and settlement.

## Methods

### Sampling

Data presented here were obtained in 2009 from 34 informants to explore Mexican immigration and settlement in Columbus, Ohio. The sample represents three distinct areas of the Columbus metropolitan region, specifically the Hilltop neighborhood on the city’s southwest side; Whitehall on the city’s east side and Worthington on the city’s north side, both independent municipalities [44]. This original sample included 17 men and 17 women, ages 18 to 62 years (mean = 35 years). All informants originally migrated from Mexico. Length of residence in Columbus varied (range 5–30 years). Most participants (*N* = 30) had settled elsewhere in the US before moving to Columbus. In general, these migrants came from both rural and urban settings in the southern Mexican state of Oaxaca. Most report they quickly found jobs in service and light industry in the US although they had little education, advanced training, or work experience. Most participants stated they had crossed the border to access higher hourly wages in the US and rendered their decision to migrate in blatant economic terms.

Thirty-four individuals were interviewed. To document physical outcomes, we asked 28 of those 34 to participate in a series of anthropometric and physiological measurements and to complete self-assessments of their health and well-being. These 28 informants self-reported perceptions of their own health and well-being and allowed us to measure physiological biomarkers. Measured biomarkers included systolic and diastolic blood pressure, blood glucose, weight, height, and four skinfolds. Several of these physiological measures are established secondary modulators of stress response and biomarkers of stress-related physiological dysregulation [45–48]. To assess participants’ perceptions of their current physical and mental health status and well-being, each completed a series of self-report questions from the Medical Outcome Study 36-Item Short-Form Health Survey [49].

### Biomarkers

All anthropometric assessments followed standard protocols as published by Lohman et al. [50] and were completed by the same researcher. Anthropometric measurements were completed while participants were wearing light clothing. Height was quantified using a GPM® Anthropometer. While each participant stood in the proper anthropometric position described by Lohman et al. [50], their height was measured twice and recorded to the nearest millimeter. The average of these two measurements is used as our measure of height. Body weight was measured twice to the nearest kilogram using a Health-O-Meter® portable scale. The average of these two measurements is used as our measure of weight. Body mass index (BMI = weight (kg)/height (m^2^)) was determined from the average weight and height measures.

Systolic and diastolic blood pressures were measured according to Systolic Hypertension in the Elderly Program protocols using a Baumanometer® mercury sphygmomanometer, a Littman® stethoscope, and appropriately sized cuffs while participants were seated [6]. Blood pressures were measured three times with 5-min intervals between measures. This protocol also conforms to guidelines established by the Seventh Report of the Joint National Committee on Prevention, Detection, Evaluation, and Treatment of High Blood Pressure published by the American Heart Association [51]. The average of all three measurements is used for analyses.

Skinfolds reflect body composition and fat patterning and are significantly associated with chronic disease morbidity, including coronary artery disease, stroke, and diabetes. Skinfolds were measured at the triceps, calf, subscapular, and suprailiac locations as specified by Lohman et al. [50] on the skin or with light coverings using a Lange® Skinfold Caliper.

Blood glucose was assessed using a Glucometer3 that was tested for reliability on a daily basis [52]. A random glucose level either at or above 125 mg/dl was used to determine hyperglycemia.

### Self-reported health

Questionnaires, consent forms, and instructions for participants were presented in Spanish by a native speaker. A self-report questionnaire was used to elicit information on age, sex, years lived in Columbus and the US, personal and family medical history, lifestyle, social activities, and health using the SF-36 health survey [53]. Written in English, the questionnaire was translated to Spanish by a native speaker. Participants responded mainly in Spanish. The SF-36 requires participants to self-administer a 36-item questionnaire to describe their self-perceptions of their own physical health and emotional well-being in relation to normal social activities with family, friends, neighbors, and co-workers. Informants could score a maximum of 24 points based upon their responses. A low total score indicates the informant perceived her or himself to be in poor health, while a high score indicates a perception of good health.

## Results

### Blood pressure

Following internationally established clinical guidelines [51], over half of our 28 informants had normal blood pressure (Table 1). Systolic blood pressure averaged 114.4 mmHg (sd = 12.7, range 90–148 mmHg). Diastolic blood pressure averaged 78.3 mmHg (sd = 9.9, range 60–92 mmHg). Of the 28 persons measured, 6 (21%) showed a blood pressure level indicative of hypertension.

**Table 1:** Distribution of sample by systolic (SBP) and diastolic (DBP) blood pressure (*N* = 28)

Systolic (mmHg)	*N*	Diastolic (mmHg)	*N*
90–120	22	60–80	17
121–139	5	81–89	8
140–159	1	90–99	6
≥ 160	0	≥ 100	0
Total	28	Total	28

### Glycemia

Glycemia averaged 142 mg/dl (sd = 55.3; range 83–289 mg/dl) among our informants. For the majority of our sample (9 men/12 women) blood glucose fell within the normal range (70–125 mg/dl; Table 2). However, 25% of the sample were hyperglycemic according to international standards (glycemia ≥ 126 mg/dl) [54].

**Table 2:** Distribution of sample by glucose level and sex (*N* = 28)

Category	Indication	Male (*n = 13*)	Female (*n = 15*)
70 mg/dL and below	Lower-than-normal (hypoglycemic)	0	0
70–125 mg/dL	Normal	9	12
126 mg/dL and above	Higher-than-normal (hyperglycemic)	4	3

### Anthropometrics

In 1988 and later, the World Health Organization suggested that BMI (kg/m^2^) is a useful index of obesity-related health risks, recommending specific international guidelines for assessing body habitus. According to WHO [55], a BMI between 18.5 and 25.0 kg/m^2^ reflects normal body habitus, while a BMI between 25 to 30 kg/m^2^ is considered overweight and one of 30 or over indicates obesity. The distribution of BMI observed in this sample is broad, 19.5–41.2 kg/m^2^, averaging 29.1 kg/m2 (sd = 5.7). Forty-three percent show a BMI between 25 and 30 kg/m^2^. Thirty-six percent show a BMI of 30 kg/m^2^ or more, a group including equal percentages of men and women.

We measured skinfold thickness at the triceps, suprailiac, subscapular, and medial calf to estimate subcutaneous fat deposits. As with BMI, skinfolds show a broad range of variation (Table 3). For example, the subscapular skinfold ranged from 10 to 40 mm and averaged 24.5 mm (sd = 7.30 mm: Table 3). Weight and height also varied widely among our informants. Minimum weight was 45.4 kg, while the maximum was over twice the minimum, 105.7 kg. Weight averaged 74.8 kg (sd = 17.4 kg). Minimum height was 141.0 cm, the maximum was 179.0 cm with a mean of 162.2 cm (sd = 10.8 cm). Both the tricep and subscapular skinfold average measurements were well above the US national average values, 14.9 mm and 20.2 mm, respectively (Table 3) [56].

**Table 3:** Anthropometric measurements (*N* = 28)

Biomarker	Min	Max	Mean	SD
Weight (kg)	45.4	105.7	74.8	17.4
Height (cm)	141.0	179.0	162.2	10.8
BMI (kg/m^2^)	19.5	41.2	29.1	5.7
Waist circumference (cm)	69.0	135.0	98.5	15.5
Hip circumference (cm)	84.0	136.0	106.7	12.4
Tricep (mm)*	12.0	39.0	24.1	7.0
Subscapular (mm)*	10.0	40.0	24.5	7.3
Suprailiac (mm)*	15.0	43.0	27.0	6.3
Medial calf (mm)*	13.0	35.0	22.0	5.8

*Indicates skinfold thickness measurements

### Perceptions of health status

Our informants self-assessed their well-being and health by responding to a series of Likert-type questions. In response to the question: Would you say your health is as follows: excellent = 1, good = 2, fair = 3, and poor = 4; 14.3% responded excellent and 39.3% good, suggesting the majority (53.6%) perceive themselves to be in good or better health. However, 39.3% responded fair and 7.1% as poor health (Table 4), almost half of all respondents (46.4%). Those indicating fair or poor health frequently noted experiencing persistent bodily pain along with back and knee pain associated with their work. Problems with chronic pain also were attributed to Ohio’s winter climate and the respondents’ inability to adapt to the cold.

**Table 4:** Self-assessed health status (*N* = 28)

Rank	Health status	*N*	Percentage
1	Excellent	4	14.3
2	Good	11	39.3
3	Fair	11	39.3
4	Poor	2	7.1
	Total	28	100.0

### Self-reported health status

Based upon the SF-36 [49], we probed how our informants perceived their health. The total possible score on the SF-36 is 24 points. Responses ranged from 6 (poor health status) to 21 points (good health status) with a mean of 10.2 (sd = 4.3), indicating a relatively low self-perception of well-being overall within this sample. Nearly a third (32%) of our informants scored seven or lower, indicating a frequent perception of extremely poor health. Another third of our sample fell between 8 and 11. In their narratives and discussions, informants often attributed their perceived poor health to economic changes in the US, living in the Midwest, job loss, and limited access to health services. Several informants were undocumented immigrants; they noted their lack of ability to access health, employment, and life style opportunities because of their legal status.

## Discussion

Our different sources of data do not produce clear associations with one another: this is not an original observation. Others have failed to observe significant associations among self-reports, narratives, and clinical measures of health among migrants also [15, 16]. Consequently, many have considered these domains separately [16, 23, 24]; however, we suggest viewing them as a system. Independently, participant narratives were overwhelmingly positive. Conversely, assessments of biomarkers revealed poor health profiles among a large proportion of our sample. Informants also reported experiences of discrimination, while tending to self-report their health as good. Although small, in this sample, neither narratives nor self-reports of health are valid proxies for participants’ clinically assessed physiological health. We propose people’s narratives reveal more about self-perceptions of their current circumstances and how they self-situate themselves within their current and previous environments than their health or physical well-being, thereby providing a viewpoint or mindscape [57] for organizing their migration experience.

As part of their narratives, informants often spoke of discrimination, which they viewed as something that happened outside the household. They spoke of home as a safe haven, a place to relax, speak Spanish, and enjoy family life. Discrimination and harassment were described as real and important issues confronting our informants as they moved about in the city, while at work, and to their children when they were in schools. By sharing narratives in their homes, respondents were removed from the challenges of life in the city and freely discussed and commented on experiences of discrimination and harassment. Home provided participants the space they likely required to negotiate the meanings of their experiences and think through the significance of previous events and social interactions.

Narratives are a lens through which migrants perceive, process, and relate their experiences that may influence biomarkers of stress and health outcomes. Constructing narratives of oneself is a natural and normal human practice. Narratives serve to compartmentalize hardships, organize events, feelings, and doubts and provide individuals with a sense of control over their experiences and likely promote contentment with life [58]. Although narratives are a singular source of data, they provide information across multiple domains. Narratives largely are subjective, qualitative, and personalized accounts and retellings of experiences and feelings. Similarly, self-reports of health are subjective assessments of one’s personal health relative to themselves at earlier ages and others around them. As such, they are interpretations of both how one feels and known aspects of their physiological function. In contrast, physiological biomarkers are objective, quantitative, and reproducible assessments of current function and health at a specific point in life. Qualitative assessments of health were not strongly associated with quantitative measures, suggesting narratives and self-reports are not indicative of actual somatic health and well-being in this sample. For example, while narratives generally focused on positives of their migration experiences, elevated blood pressure and hyperglycemia in a large proportion of the sample suggest they may be experiencing physiological responses to stress.

All migrants are imbedded within sociocultural systems while their physiology responds to a constellation of stressors permeating their everyday lives. We view narratives as providing a social and personal tool for coping with and managing the uncertainty, negativity, and ambiguity encountered as marginal members of their receiving societies. Differences among narratives people tell, their objectively measured biomarkers, and their self-reports of health illustrate some of the complexities characterizing their migration and settlement. This lack of coherence across different domains of perceived and assessed health likely reflects experiential variation among our informants and a general dissonance between maintenance of self-image and their experienced stressors and outcomes of migration over time. Physiological data reference measurable health outcomes, while narratives are what people live. Thus, we cannot explore them as similar data types or sets; rather, they must be contextualized in relation to one another to produce a broader picture of migrant health.

**Fig. 1:**
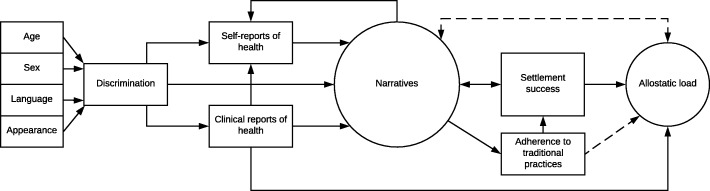
Allostatic load model incorporating self-reports of health, clinical reports of health, discrimination, and migration narratives

Based on this review and data, we propose a model integrating the various aspects of migrant health we have enumerated (Fig. 1). Our suggestion is that these domains are integrated by the narratives people compose to tell their migration stories. We suggest informants’ variable sociocultural responses (narratives), reports of discrimination, self-reports of health, and clinical biomarkers are filters through which people manage a diverse range of situations and stressors experienced by them throughout their lives. Narratives that migrants compose are used to process their circumstances and color their perceptions of their own health. Any discrimination a migrant may experience stems from the way they are perceived by others in their community. Factors that influence type and severity of discrimination include age, sex, language abilities, and appearance. Encounters with discrimination likely affect both their self-reports of health and their physiological health, conceivably through embodiment of social inequality, disproportionate economic inequality, and poor access to resources such as health care [59–61]. Therefore, discrimination begins our model as it affects all later domains (Fig. 1). Jointly, these three components influence conceptualizations of each individual’s narrative of their migration experiences. Narratives themselves also moderate allostatic load. Perceived stressors influence not only our worldview, but our responses. As narratives conceptualize experience, they moderate allostatic responses to stressors, influencing clinical assessments of physiological function. The relationship between narratives and allostatic load is represented with a dashed double arrow to symbolize the recursive relationship between perceptions, responses, and physiological health.

Additionally, clinical reports of health influence self-reported health as do narratives, although in unpredictable ways. Because self-reported health is imbedded in a social system, it is not a proxy for physiological health. Jointly, clinical and self-reports of health along with narratives interact in a complex relationship that modulates allostatic load and later life health outcomes. Based on this model, successful migration and settlement reflect adaptive, time-dependent responses to a changing multi-dimensional environment and the construction of a successful migration narrative. Settlement success is also moderated by a migrant’s adherence to their traditional cultural practices during their adjustment to a new society. This can even manifest as migrants coopting aspects of the local culture within a framework of their traditional cultural practices to achieve social mobility in a new country [62]. Narratives, self-reports, and clinical assessments each provide a unique data set, and therefore, different views of the processes and outcomes of migration as social and physiological adaptations continue over time. By determining how these different domains interact and individuals differentially respond to stressors of migration, we will better understand how individuals construct their lives and maintain physiological balance within a complex nexus of social, ideological, and physiological stressors related to migration. Dissonance across these different data sources illustrates the complexity of factors accompanying the migrant experience and why conflicting results often arise from different studies.

Migration is a physiological and cultural process that includes multiple complex social, behavioral, dietary, and physiological interactions. As a complex process, migration and the stress that come with it are defined and managed through the narratives people tell, their physical abilities and perceptions, individual health status, and perceptions of their own well-being. To capture the sociocultural as well as the physical realities of migration, it is necessary to hear narratives of migration from informants while also measuring physiological biomarkers and determining how informants assess their own health and well-being. Connections between the narratives people share, biomarkers, and self-reports of health may not always be obvious, but should be viewed as indicative of the individual’s dynamic responses to migration.

## Conclusions

We propose variation among different measures of migration stress and lack of obvious associations among these measures are to be expected as a consequence of variable adaptive responses and strategies by different migrants to their own issues. Narratives, biomarkers, and self-assessments of health measure different aspects of the migration experience. Taken together, these measures bridge ethnographic and physiological data, define the dynamic complexities of migration, and reveal the multiple ways by which people adapt and cope with the difficult and multi-dimensional challenges that come with migration. People may feel or experience something much different from what they say, and the inconsistencies between biomarkers, self-reports, and narratives may reflect this discrepancy [63, 64].

Incorporating physiological measures of allostatic load with narratives of migration and settlement will aid in unraveling the complex relationships that define acculturation and its influences on health. Mexico’s residents, like those from other countries, are not homogenous and neither are Mexican migrants to the US. Although many migration stressors are ubiquitous, migrants from different regions often present unique challenges and may incorporate different buffers from stressors (e.g., demographics, local legislation, quality of education, and healthcare). Narratives of perceived stress and physiological data often are contradictory. Thus, continued exploration of regional patterns may help elucidate subtleties in how stressors of migration and locally based buffers moderate allostatic load across samples.

## Abbreviations

BMI: Body mass index
SES: Socioeconomic status
US: United States
